# Determination of $k_{Q}$ using MLC‐collimated rectangular fields for absolute dosimetry of the CyberKnife

**DOI:** 10.1120/jacmp.v16i6.5720

**Published:** 2015-11-08

**Authors:** Jacob A. Gersh, Benjamin Willett

**Affiliations:** ^1^ Department of Radiation Oncology Gibbs Cancer Center and Research Institute Greer SC USA; ^2^ Spectrum Medical Physics LLC. Greenville SC USA; ^3^ Accuray Inc. Sunnyvale CA USA

**Keywords:** CyberKnife, absolute dosimetry, MLC, TG‐51

## Abstract

Traditional CyberKnife (CK) calibration uses TG‐51, which requires $k_{Q}$ to be defined using the standard reference condition of 100 cm SSD in a 10 cm×10 cm field. Since the CK is calibrated using a 6 cm fixed‐aperture collimating cone at 80 cm SAD, the BJR‐25 method is commonly used to relate circular‐field PDDs to square‐field PDDs for $k_{Q}$ determination. Using the InCise MLC system, the CK is able to deliver rectangular fields, allowing a more direct measurement of %dd(10 cm) using conventional reference conditions. We define the PDD correction factor ($C_{\text{PDD}}$) as the ratio of %dd(10 cm) measured using CK reference conditions to that measured using standard TG‐51 reference conditions. Using four ionization chambers (A1SL, CC08, CC13, and A19), %dd(10 cm) is measured using a 6 cm fixed cone at 80 cm SSD and at 100 cm SSD using an effective 10 cm×10 cm MLC‐collimated field. These values are used to calculate $C_{\text{PDD}}$, while the latter is used to directly calculate a $k_{Q}$ value. This direct $k_{Q}$ value is then compared to values determined using the BJR‐25 method. Using the MLC system, this study demonstrates conversion between the %dd(10 cm) measured using CyberKnife reference conditions and TG‐51 reference conditions. These values provide the means for derivation of a $k_{Q}$ curve as a function of direct measurements of %dd(10 cm) using a 6 cm fixed‐aperture collimating cone at 80 cm SSD.

PACS number: 87.55.Qr

## I. INTRODUCTION

The standard method for the absolute calibration of a CyberKnife (CK) system (Accuray Inc., Sunnyvale, CA) is based on the dosimetric principles and techniques presented in TG‐51 or an equivalent dose‐to‐water dosimetry protocol (for example, IAEA TRS‐398).^1^, ^2^ With respect to calibration techniques, the major difference between a standard linear accelerator (linac) and the CK is the reference conditions upon which absorbed dose is determined. The CK system does not use the traditional TG‐51 reference conditions of a 10 cm×10 cm field at 100 cm SSD/SAD. Instead, the CK is calibrated to deliver 1.00 cGy/MU at 80 cm SAD at a depth of 1.5 cm using a 6 cm fixed‐aperture collimating cone (a diameter which is defined 80 cm from the source). The major repercussion of this change in reference conditions is that the beam quality conversion factor, $k_{Q}$, is only defined for a 10 cm×10 cm square field at 100 cm SSD. The current vendor‐recommended procedure for absolute calibration includes determining $k_{Q}$ using a process of equivalent square field size conversions, and field‐size specific %dd(10 cm) conversion through the use of BJR‐25.^(3)^ The vendor mentions that, for users with the optional InCise Multileaf Collimator (MLC) system, the PDD could be directly measured.^(4)^ Using the larger field sizes afforded by the MLC system, this study provides the CyberKnife physicist with the means to perform an accurate conversion between the %dd(10 cm) measured with a 6 cm fixed‐aperture collimating cone at 80 cm SSD and that which is measured with a 10 cm×10 cm square field at 100 cm SSD.

The beam quality conversion factor, $k_{Q}$, is used to convert an ionization chamber's calibration factor in a ${\;}^{60}\text{Co}$ beam for use in a beam of quality Q. This factor, which takes into account the difference in absorbed dose to water between the calibration field and the clinical field, is a function of beam quality and is chamber‐dependent. The report of TG‐51 provides $k_{Q}$ values for various chamber and beam quality combinations, in both plot and tabular formats. An addendum to TG‐51 provides an updated and expanded list of detectors, and introduces coefficients for a polynomial fit to Monte Carlo‐derived $k_{Q}$ curves.^5^, ^6^, ^7^ An important caveat to the use of $k_{Q}$ values is that they are based on percent depth‐dose values for 10 cm×10 cm fields at 100 cm SSD (subsequently referred to as the "TG‐51 reference condition"). The recommendations of the 2014 addendum also use the TG‐51 reference condition.^(7)^ Since calibration of the CK is performed at 80 cm SAD with a 6 cm fixed‐aperture collimating cone (subsequently referred to as the "CK reference condition"), the authors of the addendum explicitly state that the concepts cannot be applied directly to the calibration of the CyberKnife. Therefore, corrections must be applied in order to account for the CyberKnife's unique reference conditions.

In choosing the reference condition of the CK system, the maximum field size is selected, which limits the beam to a maximum of 6 cm in diameter (5.4 cm equivalent square field size at 80 cm SSD). Many CK systems have both the fixed collimator housing and the variable‐aperture (Iris) collimator housing; however, all CK systems have the fixed collimator housing. By basing reference conditions on the collimation system ubiquitous to all CK systems, greater standardization of calibration technique is achieved. Additionally, since there cannot be variability in the diameter of the fixed collimator (such as is the case for the variable‐aperture system), consistency in reference conditions is maintained. Since CK users are restricted to the use of circular fields, for TG‐51 purposes, the vendor suggests determining $k_{Q}$ by equating TG‐51 reference conditions to CyberKnife reference conditions via field‐size specific %dd(10 cm) conversion through the use of BJR‐25 data.^(3)^

As Sharma et al.^(8)^ describes, $k_{Q}$ determination using the BJR approach begins with measuring the PDD at a depth of 10 cm using the 6 cm fixed‐aperture collimating cone at 100 cm SSD. This field size is converted to an equivalent square field size of 6.75 cm×6.75 cm.^(9)^ This field would be equivalent to 5.4 cm×5.4 cm at 80 SSD.^(10)^ Through interpolation between curves of PDD versus nominal energy, an equivalent %dd(10 cm) can be determined for the field size of 6.75 cm×6.75 cm. These techniques of absolute calibration have widespread acceptance in the CK community, and have been verified using TLDs and OSLDs at calibration laboratories such as the IROC and MD Anderson. However, these methods use BJR values which were based on jaw‐collimated beams, which have a different amount of scatter, transmission, and leakage associated with their use. Similarly, the scatter, transmission, filtration, and leakage of a CyberKnife will differ than those measured in a conventional linac.

Effective square field data in this study are acquired from beams collimated using the InCise multileaf collimation system (Accuray Inc.). Available only on the CyberKnife M6 system, in addition to fixed‐ and variable‐aperture collimators, the InCise system extends the field size capabilities of the CK, giving the CK system the ability to provide a 12 cm×10 cm field (where the 12 cm field is in the direction of leaf motion).^11^, ^12^ This field size is defined at 80 cm SAD. The 82‐leaf system (41 leaf pairs) consist of 2.5 mm wide, 80 mm thick tungsten leaves as projected at the 80 cm definition point.

Since the current study is be representative of the characteristic beam energy of the specific CyberKnife M6 system used, subtle intermachine differences in beam energies may result in the subsequent differences between the measured PDD values using the two reference conditions. By adjusting the system's injector current, and repeating the study, we are able to provide data for a range of beam energies so as to encompass the beam energies of the CyberKnife systems in clinical use, which typically yield %dd(10 cm) values between 60.5% and 61.5%.

## II. MATERIALS AND METHODS

**Figure 1:**
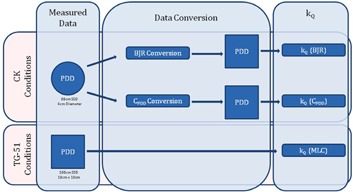
Flow chart summarizing the three methods of determining $k_{Q}$ used in the current study; the BJR method, the $C_{\text{PDD}}$ method, and the MLC method. The "conditions" refer to the conditions in which the PDD is acquired; either CK conditions (80 cm SSD using a 6 cm fixed‐aperture collimating cone), or TG‐51 conditions (100 cm SSD using a 10 cm×10 cm field).

Represented in Fig. 1, $k_{Q}$ is determined using three methods: the traditional BJR method, the MLC method (where %dd(10 cm) values are directly acquired using the TG‐51 reference condition), and a method which converts %dd(10 cm) values from the CK reference condition to values that would be acquired using the TG‐51 reference condition. Three sets of %dd(10 cm) values are acquired with four detectors at five different energies, each acquired using different combinations of collimation width, collimation shape, and SSD. The ionization chambers used in the current study are shown in Table 1. These chambers are chosen based on their utility for absolute calibration, as well as the availability of current $k_{Q}$ data. More specifically, all four chambers are mentioned in the 2014 addendum to the TG‐51 report.^(7)^ The chambers used in this study are very similar in construction and composition, mainly differing in cavity dimensions (and subsequently, cavity volume).

For each chamber, a %dd(10 cm) is acquired at 80 cm SSD using the 6 cm fixed‐aperture collimating cone. Since the vendor recommends that the calibration be performed using fixed collimators as opposed to using the variable‐aperture Iris collimator, PDD measurements are acquired using the 6 cm fixed‐aperture collimating cone. The %dd(10 cm) is determined by taking the ratio of the reading acquired at a shifted depth of 10.18 cm (10.12 cm for the A1SL) to the maximum reading acquired along the central axis. It is important to note that the maximum reading is not necessarily that which is measured at the shifted depth near 1.5 cm; importance is placed on more accurately characterizing the beam energy. Next, a similar measurement is performed with the InCise collimator using an effective field of 10 cm×10 cm at 100 cm SSD.

**Table 1:** Characteristics of the chambers used in the current study

*Chamber Type*		*Volume (cc)*	*Cavity Radius (mm)*	*Collector Length (mm)*
Exradin	A1SL	0.053	2.0	4.4
IBA	CC08	0.08	3.0	4.0
IBA	CC13	0.13	3.0	5.8
Exradin	A19	0.62	3.0	21.6

The InCise system places a leaf at the central axis, and since the leaf width is 2.5 mm, the system cannot produce a true 10 cm×10 cm. The MLCs are set to give a field size of 7.77 cm×8.25 cm (at 80 cm SSD), yielding a field size of 9.71 cm×10.31 cm, which is a rectangular field with equivalency to a 10 cm×10 cm field at 100 cm SSD. As a test to ensure the equality of a 10 cm×10 cm field to a 9.71 cm×10.31 cm field, PDD curves were acquired for both field sizes from a 6 MV unflattened beam of a Varian TrueBeam (Varian Medical Systems, Palo Alto, CA) at 100 cm SSD in a water tank using a CC13 (IBA Dosimetry, Schwarzenbruck, Germany). The two curves were indiscernible upon superimposition, with PDD values for the 10 cm×10 cm field being larger by less than 0.05% on average.

Since the energy of the CyberKnife system's 6 MV beam will vary slightly from system to system, a vendor‐employed physicist participated in this study and varied the energy of the beam over a range that encompasses a realistic range of energies found in clinical CyberKnife systems. With data acquired across these discrete energy steps, an energy‐dependent analytical expression may be formulated, providing the CK physicist with a %dd(10 cm) conversion value specific to the machine's energy. In this study, the beam energy is characterized by the %dd(10 cm) measured at 80 cm SSD using a 6 cm fixed‐aperture collimating cone — a directly measurable quantity.

For benchmark against current techniques, the vendor‐recommended method (described by Sharma et al.^(8)^) is used to determine $k_{Q}$. Therefore, in addition to the aforementioned measurements, for each energy and detector, an additional %dd(10 cm) is determined at 100 cm SSD using the 6 cm fixed‐aperture collimating cone.

Measurements are acquired using a Max 4000 electrometer (Standard Imaging Inc, Madison, WI) while chambers were positioned using a Standard Imaging DoseView 1D water tank. Prior to measurements, field sizes were verified using radiochromic film.

All subsequent derivations are based on the assumption that %dd(10 cm) is equal to $\%\text{dd}(10\;{\text{cm})}_{x}$. The recently published addendum to TG‐51 suggests that, for all flattening filter‐free beams, a lead foil be used for the determination of $\%\text{dd}(10\;{\text{cm})}_{x}$.^(7)^ The current study uses the traditional TG‐51 cutoff, where %dd(10 cm) is equal to $\%\text{dd}(10\;{\text{cm})}_{x}$ for energies under 10 MV.^(1)^ At the time of publication, no CyberKnife‐specific study on electron contamination in an unflattened beam has been performed. The reader should consider, if applicable, the application of the results of such studies to the methods presented herein.

We define a value $C_{\text{PDD}}$ which is the ratio of %dd(10 cm) measured with a 6 cm fixed‐aperture collimating cone at 80 cm SSD to the %dd(10 cm) measured with a 10 cm×10 cm square field at 100 cm SSD. Since an effective square field is used, the relation is as follows:

(1) $$C_{PDD}=\left(\frac{\%dd{\left(10\right)}_{80cm\;SSD}^{\phi 6cm}}{\%dd{\left(10\right)}_{100cm\;SSD}^{10cm\times 10cm}}\right)=\left(\frac{\%dd{\left(10\right)}_{80cm\;SSD}^{\phi 6cm}}{\%dd{\left(10\right)}_{100cm\;SSD}^{9.71cm\times 10.31cm}}\right)$$

Therefore, $k_{Q}$ will be a direct function of the %dd(10 cm) measured at 80 cm SSD using the 6 cm fixed‐aperture collimating cone, as follows:

(2) $$k_{Q}(\%dd{\left(10\right)}_{100cm\;SSD}^{10cm\times 10cm})=k_{Q}(\%dd{\left(10\right)}_{100cm\;SSD}^{9.71cm\times 10.31cm})=k_{Q}\left(\frac{\%dd{\left(10\right)}_{80cm\;SSD}^{\phi 6cm}}{C_{PDD}}\right)$$

## III. RESULTS

**Figure 2:**
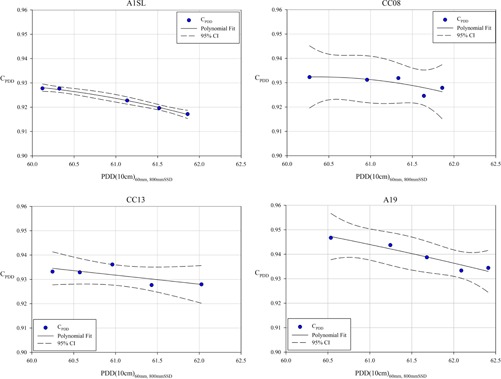
The percent depth‐dose correction factor ($C_{\text{PDD}}$) calculated for each detector at each energy evaluated during the current study. The energy is quantified by the directly measured %dd(10 cm) value acquired using a beam collimated using a 6 cm fixed‐aperture collimating cone at 80 cm SSD. Also shown are second‐order polynomial curve fits to the data, their coefficients shown in Table 2. The 95% confidence interval is also overlaid in each plot.

**Figure 3:**
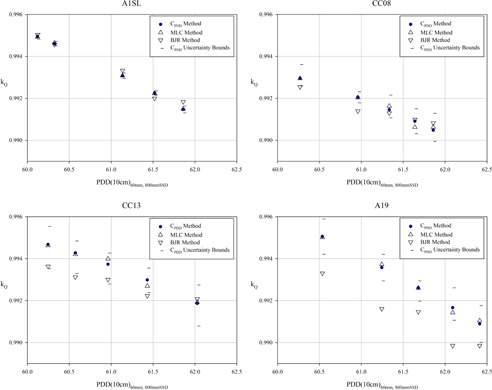
For each detector and energy evaluated in the current study, $k_{Q}$ is calculated using %dd(10 cm) values acquired using the $C_{\text{PDD}}$ method, the directly measured MLC method, and the derived BJR method. The energy is quantified by the directly measured %dd(10 cm) value acquired using a beam collimated using a 6 cm fixed‐aperture collimating cone at 80 cm SSD. The uncertainty bounds shown in the plots represent the 95% confidence interval of the analytical expression used to calculate $C_{\text{PDD}}$. The value of $k_{Q}$ is determined using the polynomial fit method, and the coefficient values provided in the 2014 addendum to TG‐51.^(7)^ The typical CyberKnife system will have a %dd(10 cm) between 60.5% and 61.5%.

For each detector, the percent depth‐dose correction value ($C_{\text{PDD}}$) was calculated for each energy and across these energies a second‐order polynomial curve was fit. The curves are shown in Fig. 2 along, with the 95% confidence interval overlaid in each plot. The coefficients of each fit are shown in Table 2. These conversion factors were used to convert %dd(10 cm) values measured using the CK reference condition to values representative of a measurements using the traditional TG‐51 reference condition. This value is then used to calculate $k_{Q}$ for the range of energies evaluated in the study. Figure 3 shows $k_{Q}$ values determined using the $C_{\text{PDD}}$ method and compares them the values determined using the BJR method and the MLC method. The independent values presented in these plots are the PDD at 10 cm depth, measured with a 6 cm fixed‐aperture collimating cone at 80 cm SSD. Averaged across the energies evaluated in this study, the deviation between $k_{Q}$ values calculated using the $C_{\text{PDD}}$ method and the BJR method were within measurement uncertainty for the A1SL (0.0002) and for the CC08 (0.0003). The average deviation was measured as 0.0008 for the CC13 and 0.0016 for the A19.

**Table 2:** Coefficients for the second‐order polynomial equation representing the $C_{\text{PDD}}$ for each of the detectors evaluated in the current study. The coefficients are for the form $y={\text{ax}}^{2}+\text{bx}+c$, where x is the directly‐measured %dd(10 cm) value acquired using a beam collimated using a 6 cm fixed‐aperture collimating cone at 80 cm SSD, and y is the $C_{\text{PDD}}$. These curves are shown in Fig. 2, and compared to the data which serve as the basis for this curve fit

	*A1SL*	*CC08*	*CC13*	*A19*
a	−1.5568E–03	−2.7248E–03	−2.3242E–03	−4.0891E–04
b	0.18355	0.32894	0.28051	0.042693
c	−4.4801	−8.9951	−7.5299	−0.13877

## IV. DISCUSSION

Since all CyberKnife systems are calibrated using a 6 cm fixed‐aperture collimating cone (including those with the new InCise MLC system), this conversion factor allows an accurate determination of the quality conversion factor, $k_{Q}$, by relating the directly measured %dd(10 cm) at 80 cm SSD to that which would be measured with a 10 cm×10 cm field at 100 cm SSD. This technique of determining the %dd(10 cm) for a 10 cm×10 cm field at 100 cm SSD is a more direct approach as compared to the currently used technique of equating circular field sizes to effective square field sizes, and finding equivalent %dd(10 cm) values using BJR values. By providing the conversion factor as a function of a directly measurable quantity, $C_{\text{PDD}}$ can be determined specifically for the beam energy being measured (as characterized by %dd(10 cm)).

Both the BJR and the $C_{\text{PDD}}$ methods map a PDD reading in a 6 cm circular field to a PDD reading in a 10 cm×10 cm square field. An implicit assumption in the BJR method is that the spectrum of the CK beam is equivalent to the 6 MV spectrum of the beam used to generate the BJR PDD tables. In other words, it assumes that CK %dd(10 cm) as a function of field size is the same as the BJR PDD %dd(10 cm) as a function of field size. As a result of the different collimation and filtration systems on the CK and BJR machines, there is a high likelihood that the equality is not correct. Thus, an advantage of the $C_{\text{PDD}}$ method is that the PDD as a function of field size is inferred from the CK spectrum and not from another machine's spectrum. In principle, this should reduce the overall uncertainty in $k_{Q}$ calculation.

The chamber‐specific deviation between $k_{Q}$ values determined using the $C_{\text{PDD}}$ method and the BJR method increased with increasing chamber size. This is possibly attributed to higher signal and subsequent sensitivity to the differing scatter characteristics of the two beams. The difference can also be attributed to the cavity length of the detectors used in this study, where smaller deviations are noted with smaller cavity lengths and larger deviations are noted with larger cavity lengths. This can be due to the nonflat dose distribution of the central axis of the beam incident upon the detector, which causes slight underestimation at shallow depths, while increasing at larger depths, an effect which is more pronounced with increasing detector cavity length. The nonflat dose distribution was quantified in the current study by comparing the mean off‐axis ratio (OAR) across a measured profile and the active length of each detector used in this study. In a 60 cm circular field, the mean OAR was measured as 0.999 for the A1SL and the CC08, 0.944 for the CC13, and 0.988 for the A19. Profiles were acquired in a scanning tank at 800 mm SAD, at a depth of 15 mm, using a PTW TN60019 microDiamond detector (PTW‐Freiburg GmbH, Freiburg, Germany).

Finally, as corroboration of current methods, a notable result of the current study is showing that using an MLC‐collimated effective square for $k_{Q}$ determination yields a value close to that of the vendor‐recommended BJR method. Though larger field sizes are attainable using the MLC system, the vendor maintains its current recommendations with respect to reference conditions and the determination of $k_{Q}$. There are several reasons for this. The majority of CyberKnife systems in use are earlier versions (thus not capable of using the InCise MLC Collimator). Also, not all M6 systems are going to be equipped with the MLC system. Regardless of the collimation accoutrements available on a CK system, every CK system will be equipped with a 6 cm fixed‐aperture collimating cone, thus extending the applicability of the current study to all CyberKnife systems.

## V. CONCLUSIONS

A modality‐specific conversion factor is derived and measured for the determination of $k_{Q}$ for CyberKnife calibration. By taking advantage of the larger field sizes of the CK's MLC system, this study provides a more direct method of $k_{Q}$ determination than currently used techniques. This conversion factor is a function of the energy of the beam, and is provided as a function of the %dd(10) measured using a 6 cm fixed‐aperture collimating cone at 80 cm SSD.

## ACKNOWLEDGMENTS

The authors would like to extend their appreciation to Alan Cohen and Warren Kilby of Accuray Inc., and Kevin Shay and Ryan DuBose of Gibbs Cancer Center and Research Institute.

## Supporting information

Supplementary Material

Supplementary Material

Supplementary Material

## References

[1] Almond P , Biggs PJ , Coursey BM , et al. AAPM's TG‐51 protocol for clinical reference dosimetry of high‐energy photon and electron beams. Med Phys. 1999;26(9):1847–70. 10505874 10.1118/1.598691

[2] Andreo P , Burns DT , Hohlfeld K , et al. Absorbed dose determination in external beam radiotherapy. IAEA Technical Report Series 398. Vienna: IAEA; 2000.

[3] Jordan TJ . Megavoltage X‐ray beams: 2–50 MV. BJR Suppl. 1996;25:62–109. 9068357

[4] CyberKnife Robotic Radiosurgery System, Physics Essentials Guide. Versions 10.x/5.x/3.x: Accuray Incorporated, Sunnyvale, CA; 2014.

[5] Muir BR and Rogers DW . Monte Carlo calculations of kQ, the beam quality conversion factor. Med Phys. 2010;37(11):5939–50. 10.1118/1.3495537 21158307

[6] Muir B , McEwen MR , Rogers DWO . Measured and Monte Carlo calculated K(Q) factors: accuracy and comparison. Med Phys. 2011;38(8):4600–09. 21928633 10.1118/1.3600697

[7] McEwen M , DeWard L , Ibbott G , et al. Addendum to the AAPM's TG‐51 protocol for clinical reference dosimetry of high‐energy photon beams. Med Phys. 2014;41(4):1501–20. 10.1118/1.4866223 PMC5148035 24694120

[8] Sharma SC , Ott JT , Williams JB , Dickow D . Commissioning and acceptance testing of a CyberKnife linear accelerator. J Appl Clin Med Phys. 2007;8(3):119–25. 10.1120/jacmp.v8i3.2473 PMC5722603 17712305

[9] Day MJ and Aird EG . The equivalent field method for dose determinations in rectangular fields. BJR Suppl. 1996;25:138–51. 9068361

[10] Alfonso R , Andreo P , Capote R , et al. A new formalism for reference dosimetry of small and nonstandard fields. Med Phys. 2008;35(11):5179–86. 19070252 10.1118/1.3005481

[11] CyberKnife M6 Series Technical Specifications. Sunnyvale, CA: Accuray Inc.; 2013.

[12] Fahimian B , Soltys S , Xing L , Gibbs I , Chang S , Wang L . Evaluation of MLC‐based robotic radiotherapy. Med Phys. 2013;40(6):344.

