# Supplementary figures and images for: Determination of kQ using MLC‐collimated rectangular fields for absolute dosimetry of the CyberKnife
Source: J Appl Clin Med Phys. 2015 Nov 8;16(6):273–80. doi: 10.1120/jacmp.v16i6.5720 (PMC5690991; doi:10.1120/jacmp.v16i6.5720)

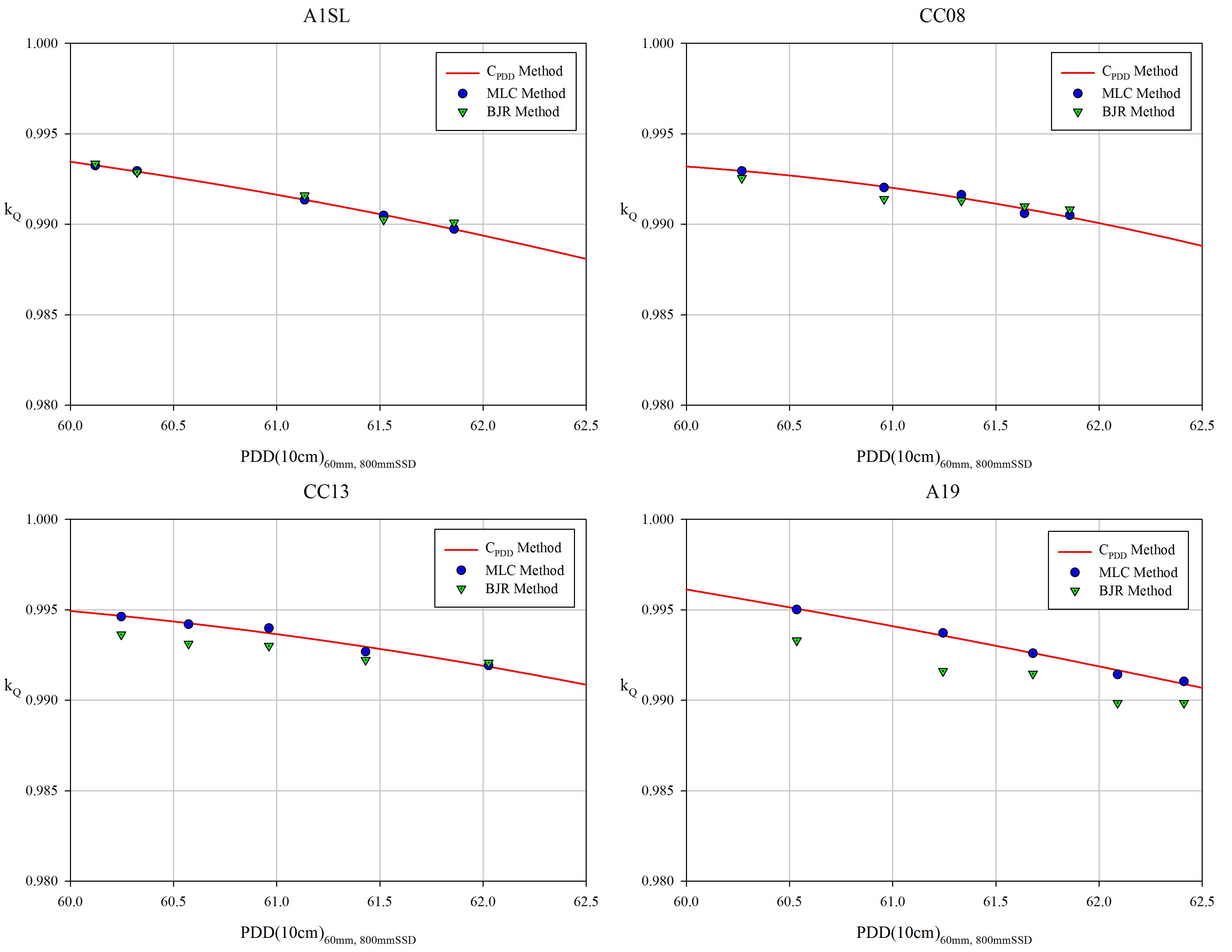

Supplement: Supplementary file 1 — Supplementary Material [file ACM2-16-273-s001.jpg]

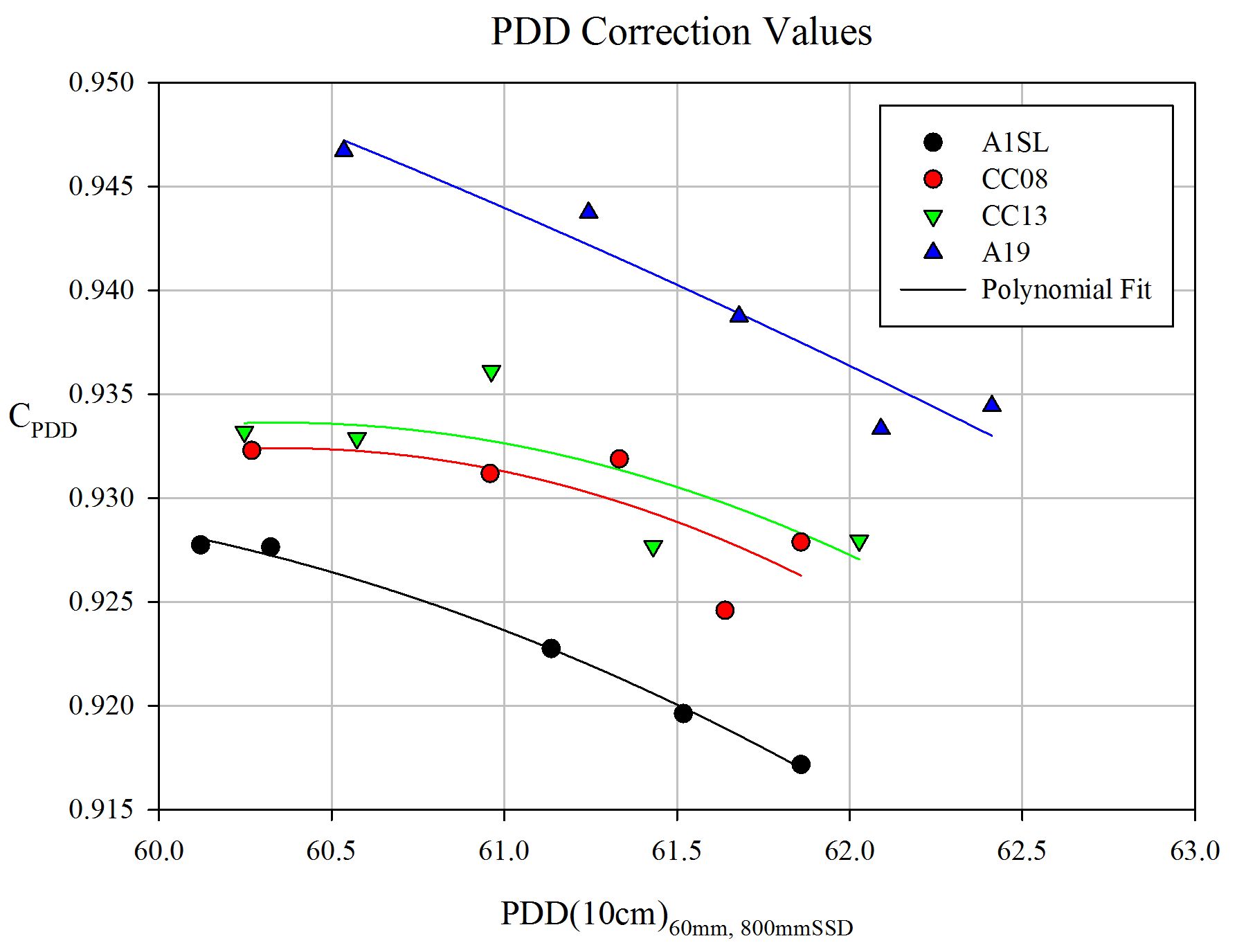

Supplement: Supplementary file 2 — Supplementary Material [file ACM2-16-273-s002.jpg]
