# Supplementary material for: Determination of kQ using MLC‐collimated rectangular fields for absolute dosimetry of the CyberKnife
Source: J Appl Clin Med Phys. 2015 Nov 8;16(6):273–80. doi: 10.1120/jacmp.v16i6.5720 (PMC5690991; doi:10.1120/jacmp.v16i6.5720)
Supplement: Supplementary file 3 — Supplementary Material [file ACM2-16-273-s003.doc]

k_Q_ determination using MLC-collimated rectangular fields

for absolute dosimetry of the CyberKnife

Jacob A. Gersh and Benjamin Willett

Jacob A. Gersh

*Gibbs Cancer Center and Research Institute, Greer, SC*

*and*

*Spectrum Medical Physics, Greenville, SC*

*jgersh@gibbscc.org*

Benjamin Willett

*Accuray Inc., Sunnyvale, CA*

*bwillett@accuray.com*

Running Title: kQ determination for CyberKnife

Corresponding author: Jacob A. Gersh, Gibbs Cancer Center and Research Institute – Pelham, 2759 S

Hwy 14, Greer, SC 29651, USA; phone: (864) 530 6400; fax: (864) 849 9710; email: jgersh@gibbscc.org
